# Optimization of the Reconstruction Settings for Low-Dose Ultra-High-Resolution Photon-Counting Detector CT of the Lungs

**DOI:** 10.3390/diagnostics13233522

**Published:** 2023-11-24

**Authors:** Dirk Graafen, Moritz C. Halfmann, Tilman Emrich, Yang Yang, Michael Kreuter, Christoph Düber, Roman Kloeckner, Lukas Müller, Tobias Jorg

**Affiliations:** 1 Department of Diagnostic and Interventional Radiology, University Medical Center of the Johannes Gutenberg-University Mainz, 55131 Mainz, Germany; moritz.halfmann@unimedizin-mainz.de (M.C.H.); tilman.emrich@unimedizin-mainz.de (T.E.); yang.yang@unimedizin-mainz.de (Y.Y.); christoph.dueber@unimedizin-mainz.de (C.D.); lukas.mueller@unimedizin-mainz.de (L.M.); tobias.jorg@unimedizin-mainz.de (T.J.); 2 German Center for Cardiovascular Research (DZHK), Partner-Site Rhine-Main, 55131 Mainz, Germany; 3 Division of Cardiovascular Imaging, Department of Radiology and Radiological Science, Medical University of South Carolina, Charleston, SC 29425, USA; 4 Mainz Center for Pulmonary Medicine, Department of Pneumology, Mainz University Medical Center, 55131 Mainz, Germany; michael.kreuter@unimedizin-mainz.de; 5 Department of Pulmonary, Critical Care & Sleep Medicine, Marienhaus Clinic Mainz, 55131 Mainz, Germany; 6 Institute of Interventional Radiology, University Hospital Schleswig-Holstein, Campus Lübeck, 23538 Lübeck, Germany; roman.kloeckner@uksh.de

**Keywords:** photon-counting detector CT, lung, slice thickness, quantum iterative reconstruction, ultra-high resolution

## Abstract

Photon-counting detector computed tomography (PCD-CT) yields improved spatial resolution. The combined use of PCD-CT and a modern iterative reconstruction method, known as quantum iterative reconstruction (QIR), has the potential to significantly improve the quality of lung CT images. In this study, we aimed to analyze the impacts of different slice thicknesses and QIR levels on low-dose ultra-high-resolution (UHR) PCD-CT imaging of the lungs. Our study included 51 patients with different lung diseases who underwent unenhanced UHR-PCD-CT scans. Images were reconstructed using three different slice thicknesses (0.2, 0.4, and 1.0 mm) and three QIR levels (2–4). Noise levels were determined in all reconstructions. Three raters evaluated the delineation of anatomical structures and conspicuity of various pulmonary pathologies in the images compared to the clinical reference reconstruction (1.0 mm, QIR-3). The highest QIR level (QIR-4) yielded the best image quality. Reducing the slice thickness to 0.4 mm improved the delineation and conspicuity of pathologies. The 0.2 mm reconstructions exhibited lower image quality due to high image noise. In conclusion, the optimal reconstruction protocol for low-dose UHR-PCD-CT of the lungs includes a slice thickness of 0.4 mm, with the highest QIR level. This optimized protocol might improve the diagnostic accuracy and confidence of lung imaging.

## 1. Introduction

High-resolution computed tomography (HRCT) is now the primary method for visualizing lung tissue, but traditional energy-integrating detector computed tomography (EID-CT) has limitations, such as poor depiction of intricate lung details due to spatial resolution constraints. This can impact diagnostic confidence and clinical judgments [1,2,3,4].

Photon-counting detector computed tomography (PCD-CT) offers improved spatial resolution and noise characteristics, enhancing lung tissue visualization even at lower radiation doses [5,6]. Unlike EID-CT, PCD-CT directly transforms X-ray photons into electronic signals, reducing electronic noise [7].

In thoracic imaging, reduced noise with PCD-CT improves the assessment of interstitial and alveolar lung conditions, which is crucial for detecting subtle changes indicating disease progression [8]. PCD-CT can minimize radiation exposure while maintaining image quality, which is especially important in lung cancer screening with HRCT [9]. In the ultra-high-resolution (UHR) setting, PCD-CT allows for a 1024 × 1024 matrix and a slice thickness as small as 0.2 mm. Studies show that UHR-PCD-CT improves the depiction of anatomical lung structures, bronchial walls, vessels, and various lung pathologies compared to EID-CT [10,11,12,13,14,15,16].

Image quality depends not only on the established detectors but also on the reconstruction parameters, i.e., the reconstruction kernel, use of iterative reconstruction, slice thickness, and in-plane resolution [17]. The first clinically approved PCD-CT scanner was introduced with a novel iterative reconstruction algorithm known as quantum iterative reconstruction (QIR, Siemens Healthineers, Forchheim, Germany), which has four strength levels (QIR-1 to QIR-4) and is specifically designed to match the hardware and software needs of the PCD-CT system. In a previous study, QIR-3 was identified as the optimal level for UHR-PCD-CT of the lungs, using a 1.5 mm slice thickness and the sharpest available lung kernel (Bl64) [18]. In a subsequent study, slice thicknesses were varied to the submillimeter range, while applying different sharpness levels of the lung kernel [19]. There, the best image quality was observed in the reconstruction with a 0.4 mm slice thickness using the Bl64 kernel. We hypothesized that the optimal QIR level would be different in the submillimeter range, especially when combined with a low-dose tin filtration protocol.

In the present study, we investigated UHR-PCD-CT scans from patients with lung diseases. For the first time, lung image reconstructions with submillimeter slice thicknesses were combined with different QIR levels and analyzed in terms of the image quality and its expected diagnostic impact.

## 2. Materials and Methods

### 2.1. Study Population

In October 2022, 51 consecutive adult patients were examined using a first-generation PCD-CT scanner (NAEOTOM Alpha, Siemens Healthineers, Forchheim, Germany) to undergo clinically indicated unenhanced UHR-CT scans of their lungs. None of these patients were excluded due to unsuitable image quality or any other reason.

### 2.2. Imaging Protocol and Radiation Dose

All images were acquired using an ultra-high-resolution protocol (Quantum HD, Siemens Healthineers, Forchheim, Germany), including scanning with 100 kVp and additional tin filtration. As recommended in the literature [19], lung images were reconstructed using the sharpest available lung kernel, i.e., Bl64. A 1024 × 1024 matrix was applied, with a fixed field-of-view of 330 × 330 mm^2^, resulting in a constant in-plane resolution of 0.32 × 0.32 mm^2^. Images were calculated using a slice thickness of 0.2 mm, 0.4 mm, and 1.0 mm. We applied the three highest levels of the quantum iterative reconstruction algorithm: QIR-2; QIR-3; and QIR-4. The clinical reference image was generated with 1.0 mm slice thickness and QIR-level 3 (1.0 mm QIR-3). Eight additional reconstructions were calculated: 0.2 mm QIR-2; 0.2 mm QIR-3; 0.2 mm QIR-4; 0.4 mm QIR-2; 0.4 mm QIR-3; 0.4 mm QIR-4; 1.0 mm QIR-2; and 1.0 mm QIR-4. The image quality (IQ) level was set to 13, which corresponded to a system-independent image quality definition and determined the tube current-time product [18]. Table 1 summarizes detailed information about the acquisition and reconstruction parameters.

For each scan, the CT dose index (CTDI_Vol_) and dose length product (DLP) were obtained from the patient-dose report. The effective dose was calculated by multiplying the DLP by the conversion factor designated for chest CT of adults at 100 kVp (0.0144 mSv/mGy × cm) [20].

### 2.3. Image Noise

For each image, the image noise was determined by one board-certified consultant radiologist with 15 years of experience, using an area-weighted mean of the standard deviations measured in three regions of interest (ROIs) on the level of the trachea between the aortic arch and the carina. Two of these ROIs had a 3 cm diameter and were placed in the surrounding air in the left and right top corners of the images. The remaining ROI had a 1 cm diameter and was located concentric in the trachea. The ROIs were placed manually using the institutional picture archiving and communication system (PACS: Sectra^®^, Linköping, Sweden). The determination of the noise in the air areas of the image is based on a previous study, which justified this method as a fast and robust image noise assessment [21].

### 2.4. Qualitative Image Analysis

Image quality was independently evaluated by three raters, two board-certified consultant radiologists with 15 and 7 years of experience and one resident with 4 years of experience. Analogous to a previous study [19], the raters assessed delineation of anatomical structures and conspicuity of pathologies in the eight additional reconstructions compared to the clinical reference (1.0 mm QIR-3), using a 5-point Likert scale: −2 = worse, no diagnostic ability; −1 = worse, unclear effect on potential diagnosis; 0 = about the same, unclear benefit or decrement; +1 = better, unclear effect on potential diagnosis; and +2 = better, increased diagnostic ability. For this purpose, eight randomly ordered layouts, with a side-by-side arrangement of the clinical reference on the left and one of the eight additional reconstructions on the right, were presented within the institutional PACS (Sectra^®^, Linköping, Sweden). Image information was blinded for all images. Standard lung windowing settings were used (W: 1500, L: −500).

The raters evaluated the delineation of three different pulmonary structures: the 4th order bronchial wall; main pulmonary fissures; and peripheral pulmonary vessels, evaluated in the right lower lobe, approximately 2 cm from the pleural surface.

To evaluate the conspicuity of pulmonary pathologies, the three raters analyzed the images three weeks before the quality assessment to identify the following groups of pathologies: lung nodules (up to three per patient); bronchial pathologies, including bronchiectasis, mucus plugs, bronchial wall thickenings, and tree-in-bud patterns; emphysema and bullae; ground-glass opacities; mosaic patterns; interstitial abnormalities, including interlobular and intralobular septal thickenings, pleural thickenings, and dendritic calcifications; and pleural effusions. Each identified lung nodule was assigned an individual score. Each other identified group of pathologies was assigned a single overall score. In the quality analysis, conspicuity was interpreted as not only the visibility of the pathology but also the capacity for its characterization, including the examination of margins and other features.

### 2.5. Statistical Analysis

All statistical computations were performed using dedicated statistical software (R, version 4.1.1, R Foundation for Statistical Computing, Vienna, Austria). Categorical and binary baseline parameters are presented as total counts and percentages, while ordinal-scale variables are shown as medians and interquartile ranges. Interval-scale variables that exhibit a normal distribution according to the Shapiro–Wilk test are presented as means and standard deviations.

Statistical differences in noise levels were analyzed using the paired-samples Wilcoxon rank test, with Bonferroni correction for multiple comparisons. One-sample Wilcoxon signed-rank tests were used to assess statistical differences among the image quality scores. We first used a two-tailed test with a theoretical median of 0 to examine the null hypothesis that the sample is equal to the clinical reference. Next, we applied a one-tailed test with a theoretical median of +2 to test the increase in diagnostic ability. The corresponding *p*-values are denoted as *p*_0_ and *p*_+2_. A *p*-value of < 0.05 was considered statistically significant.

To test the inter-reader agreement of the qualitative image analysis, we calculated Krippendorff’s alpha. Alpha values were interpreted as follows: 0.0–0.2 indicate slight agreement; 0.2–0.4 indicate fair agreement; 0.4–0.6 indicate moderate agreement; 0.6–0.8 indicate substantial agreement; and 0.8–1.0 indicate near-perfect agreement [22,23].

## 3. Results

### 3.1. Baseline Characteristics and Radiation Doses

Table 2 presents the baseline characteristics and radiation doses of the 51 included patients.

The clinical indications justifying the unenhanced UHR-CT scans were primarily related to identifying pneumonia or therapy-related pneumonitis (over one-third of cases) and diagnosing pulmonary nodules (slightly less than one-third). In the remaining cases, UHR-CT scans were attributed to other reasons (Table 3).

### 3.2. Image Noise

**Figure 1:**
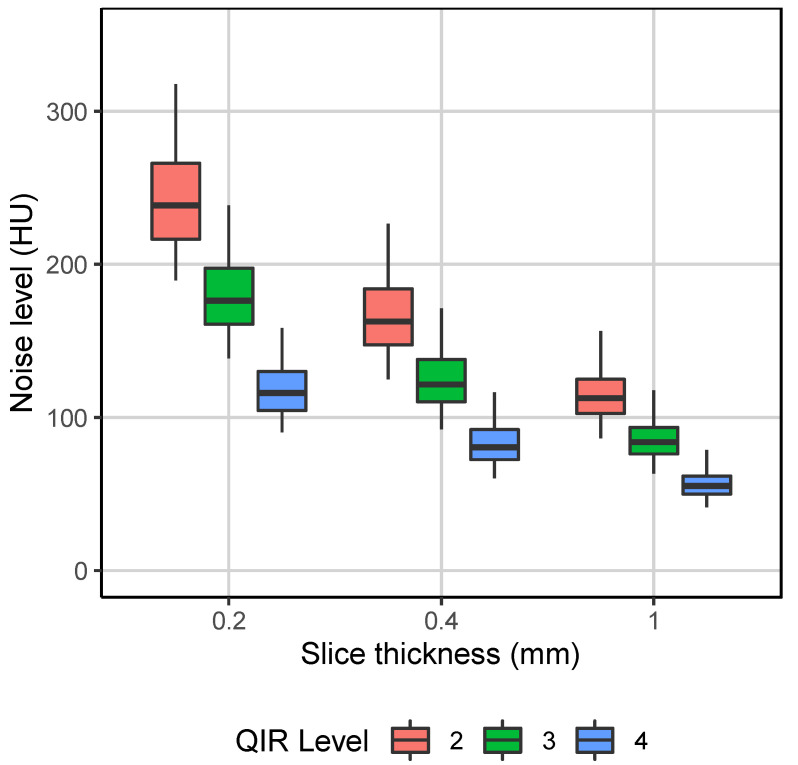
Noise level distributions of the nine image reconstructions.

Noise levels are presented in Table 4 and Figure 1. With constant QIR level, the noise levels increased by around 45% when slice thickness was reduced from 1.0 mm to 0.4 mm and by around 50% when slice thickness was reduced from 0.4 mm to 0.2 mm. These noise increases could be effectively compensated by an increment in the QIR level. For example, the noise levels were almost equal between the reconstructions between 1.0 mm QIR-3 and 0.4 mm QIR-4. The small differences in the noise levels between the following reconstructions—0.2 mm QIR-3 and 0.4 mm QIR-2; 0.2 mm QIR-4, 0.4 mm QIR-3, and 1.0 mm QIR-2; and 0.4 mm QIR-4 and 1.0 mm QIR-3—did not reach statistical significance (*p* ≥ 0.270). All other differences in noise levels were statistically significant (*p* < 0.001).

### 3.3. Qualitative Image Analysis

Evaluation of inter-reader reliability revealed substantial agreement between all raters (Alpha = 0.61), with moderate agreement between raters 1 and 2 (Alpha = 0.49) and between raters 1 and 3 (Alpha = 0.48) and a near-perfect agreement between raters 2 and 3 (Alpha = 0.82).

Table 5 summarizes the pulmonary pathologies identified for the qualitative image analysis.

#### 3.3.1. Delineation

**Figure 2:**
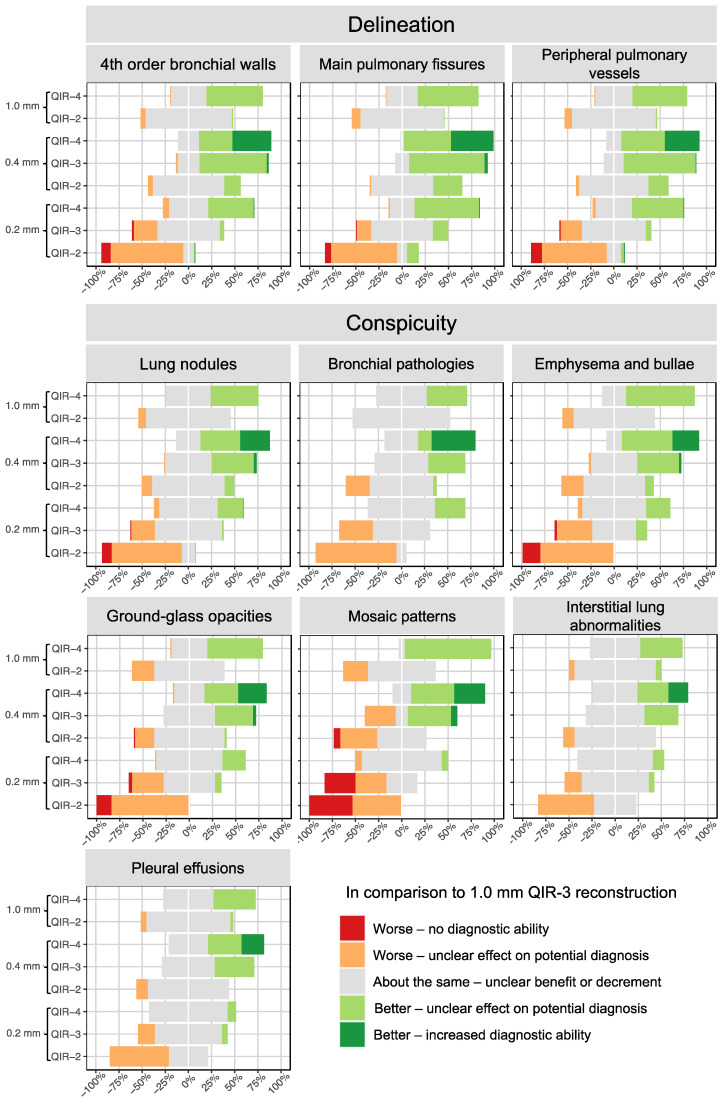
Qualitative image analysis. Delineation of three different pulmonary structures and conspicuity of different pathologies were evaluated compared to the clinical reference (1.0 mm QIR-3).

**Figure 3:**
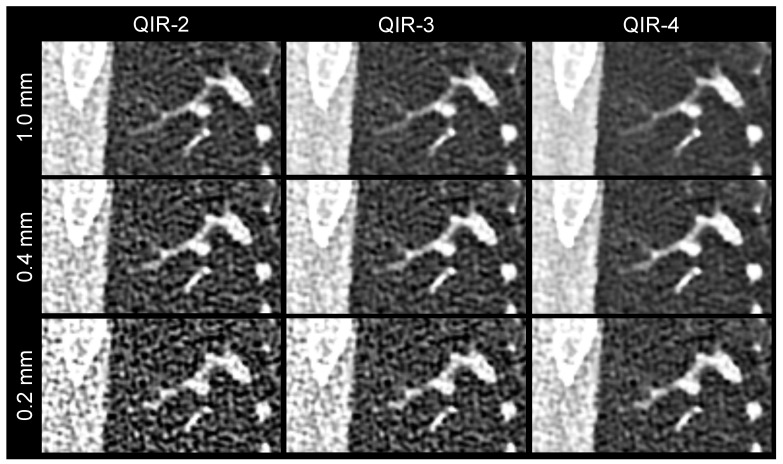
Image example showing the delineation of the peripheral pulmonary vessels. The same image detail is shown for the nine reconstructions, with 1.0 mm QIR-3 as the clinical reference. In the 1.0 mm reconstructions, the vessel borders are blurred by the partial volume effect. The 0.2 mm reconstructions are disturbed by the strong noise.

Results of the qualitative delineation analysis are shown in Table 6 and Figure 2. The delineation of the main pulmonary fissures in the reconstruction of 0.2 mm QIR-3 and the delineation of the fourth-order bronchial walls and peripheral pulmonary vessels in the reconstruction of 1.0 mm QIR-2 were not statistically distinguishable from the clinical reference (1.0 mm QIR-3). The delineation ratings of the reconstructions of 0.2 mm QIR-3, 0.4 mm QIR-2, and 1.0 mm QIR-2 were evaluated as almost equal to the clinical reference, with no clear benefit or decrement, although the differences partially reached statistical significance. All three raters observed decreased delineation in the 0.2 mm QIR-2 reconstruction, caused by a distinct increase in noise (*p*_0_ < 0.001). Delineation of the anatomical structures was found to be improved in the 0.2 mm QIR-4, 0.4 mm QIR-3, 0.4 mm QIR-4, and 1.0 mm QIR-4 reconstructions, with the 0.4 mm QIR-4 reconstruction achieving the best scores. However, none of the reconstructions yielded an improvement in diagnostic ability (all *p*_+2_ < 0.001). Figure 3 presents an example of the delineation analysis of the peripheral pulmonary vessels.

#### 3.3.2. Pathologies

The results of the qualitative analysis regarding the conspicuity of pulmonary pathologies are presented in Table 6 and Figure 2. The reconstructions 1.0 mm QIR-2 and 0.2 mm QIR-4 showed no clear benefit or drawback compared to the clinical reference (1.0 mm QIR-3).

Consistent with the results of delineation analysis, using the smallest slice thickness combined with the lowest applied QIR level (0.2 mm QIR-2) decreased the conspicuity of all pathologies due to the increased noise levels. The 0.2 mm QIR-3 and 0.4 mm QIR-2 reconstructions also showed decreased conspicuity scores due to the increased noise levels. This finding was most pronounced with regard to the mosaic patterns. Notably, due to the small number of mosaic patterns (*n* = 6), these differences should be interpreted cautiously.

Reducing the slice thickness from 1.0 mm to 0.4 mm with QIR-3 led to a slight improvement in the conspicuity scores for all pathologies (*p*_0_ < 0.001 for all pathologies except the mosaic patterns). Applying the highest QIR level (QIR-4) improved the conspicuity in the reconstructions with slice thicknesses of 1.0 mm and 0.4 mm (*p*_0_ ≤ 0.002), and this change was most pronounced with a slice thickness of 0.4 mm, where the median score was 1 for all pathologies. Notably, the raters found no expected improvement in diagnostic ability among all reconstructions (*p*_+2_ < 0.001 for all pathologies, with the exception of *p*_+2_ = 0.002 for the mosaic patterns in the 0.4 mm QIR-4 reconstruction).

**Figure 4:**
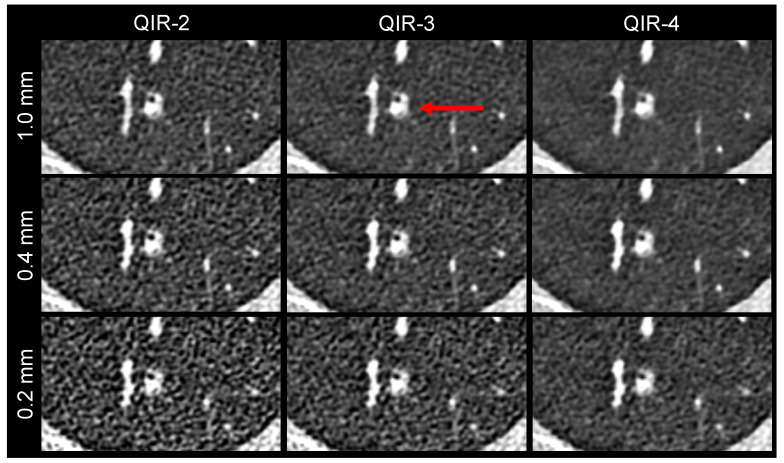
Image example, showing the conspicuity of a lung nodule. The same image detail is shown for the nine reconstructions. The nodule is marked by a red arrow in the clinical reference reconstruction (1.0 mm QIR-3).

Figure 4 presents an example of the conspicuity analysis of a lung nodule.

**Table 6:** Median scores and *p*_0_ values of the qualitative image analysis.

Slice Thickness, mm		0.2			0.4			1.0	
QIR Level		2	3	4	2	3	4	2	4
**Delineation**	4th order bronchial walls	−1 (−1 to −1) *p*_0_ < 0.001	0 (−1 to 0) *p*_0_ < 0.001	1 (0 to 1) *p*_0_ < 0.001	0 (0 to 0) *p*_0_ = 0.001	1 (0 to 1) *p*_0_ < 0.001	1 (1 to 2) *p*_0_ < 0.001	0 (0 to 0) *p*_0_ = 0.066	1 (0 to 1) *p*_0_ < 0.001
	Main pulmonary fissures	−1 (−1 to −1) *p*_0_ < 0.001	0 (0 to 0) *p*_0_ = 0.895	1 (0 to 1) *p*_0_ < 0.001	0 (0 to 1) *p*_0_ < 0.001	1 (1 to 1) *p*_0_ < 0.001	1 (1 to 2) *p*_0_ < 0.001	0 (0 to 0) *p*_0_ < 0.001	1 (0 to 1) *p*_0_ < 0.001
	Peripheral pulmonary vessels	−1 (−1 to −1) *p*_0_ < 0.001	0 (0 to 0) *p*_0_ < 0.001	1 (0 to 1) *p*_0_ < 0.001	0 (0 to 0) *p*_0_ < 0.001	1 (1 to 1) *p*_0_ < 0.001	1 (1 to 2) *p*_0_ < 0.001	0 (0 to 0) *p*_0_ = 0.008	1 (0 to 1) *p*_0_ < 0.001
**Conspicuity**	Lung nodules	−1 (−1 to −1) *p*_0_ < 0.001	0 (−1 to 0) *p*_0_ < 0.001	0 (0 to 1) *p*_0_ < 0.001	0 (0 to 0) *p*_0_ = 0.891	0 (0 to 1) *p*_0_ < 0.001	1 (0 to 2) *p*_0_ < 0.001	0 (0 to 0) *p*_0_ < 0.001	0 (0 to 1) *p*_0_ < 0.001
	Bronchial pathologies	−1 (−1 to −1) *p*_0_ < 0.001	0 (−1 to 0) *p*_0_ = 0.002	0 (0 to 1) *p*_0_ = 0.003	0 (−0.5 to 0) *p*_0_ = 0.041	0 (0 to 1) *p*_0_ = 0.001	1 (0 to 2) *p*_0_ < 0.001	0 (0 to 0) *p*_0_ = 1	0 (0 to 1) *p*_0_ < 0.001
	Emphysema and bullae	−1 (−1 to −1) *p*_0_ < 0.001	0 (−1 to 0) *p*_0_ = 0.010	0 (0 to 0.75) *p*_0_ = 0.014	0 (0 to 1) *p*_0_ = 0.117	0 (0 to 1) *p*_0_ < 0.001	1 (1 to 2) *p*_0_ < 0.001	0 (0 to 0) *p*_0_ = 0.037	1 (0.25 to 1) *p*_0_ < 0.001
	GGOs	−1 (−1 to −1) *p*_0_ < 0.001	0 (−1 to 0) *p*_0_ < 0.001	0 (0 to 0.75) *p*_0_ < 0.001	0 (0 to 0) *p*_0_ < 0.001	0 (0 to 1) *p*_0_ < 0.001	1 (0 to 2) *p*_0_ < 0.001	0 (0 to 0) *p*_0_ < 0.001	1 (0 to 1) *p*_0_ < 0.001
	Mosaic patterns	−1 (−2 to −1) *p*_0_ < 0.001	−1 (−2 to 0) *p*_0_ = 0.005	0 (0 to 0) *p*_0_ = 1	0 (−1 to 0) *p*_0_ = 0.015	1 (−1 to 1) *p*_0_ = 0.336	1 (1 to 2) *p*_0_ = 0.002	0 (−0.5 to 0) *p*_0_ = 0.072	1 (1 to 1) *p*_0_ < 0.001
	ILAs	−1 (−1 to 0) *p*_0_ < 0.001	0 (0 to 0) *p*_0_ = 0.182	0 (0 to 0) *p*_0_ = 0.072	0 (0 to 0) *p*_0_ = 0.072	0 (0 to 1) *p*_0_ < 0.001	1 (0 to 1) *p*_0_ < 0.001	0 (0 to 0) *p*_0_ = 1	0 (0 to 1) *p*_0_ < 0.001
	Pleural effusions	−1 (−1 to 0) *p*_0_ < 0.001	0 (0 to 0) *p*_0_ = 0.182	0 (0 to 0) *p*_0_ = 0.149	0 (0 to 0) *p*_0_ = 0.072	0 (0 to 1) *p*_0_ < 0.001	1 (0 to 1) *p*_0_ < 0.001	0 (0 to 0) *p*_0_ = 0.773	0 (0 to 1) *p*_0_ < 0.001

Interquartile ranges are given in parentheses. GGO: ground-glass opacity, ILA: interstitial lung abnormality.

## 4. Discussion

The present results showed that a slice thickness of 0.4 mm provided the best image quality for low-dose UHR-PCD-CT of the lungs with regard to the delineation of anatomical structures and conspicuity of pulmonary pathologies. Thinner slices, such as 0.2 mm, resulted in increased noise levels, impairing image quality without further improvements. Additionally, higher QIR levels enhanced image quality across all UHR (sub-)millimeter reconstructions, with the most favorable outcomes observed at the highest available level (QIR-4). Consequently, our findings support the recommendation of utilizing a 0.4 mm QIR-4 reconstruction protocol for low-dose UHR-PCD-CT scans of the lungs.

In a previous study, Milos et al. identified the sharpest lung kernel (Bl64) with a 0.4 mm slice thickness as the best reconstruction for UHR-PCD-CT of the lungs [19]. Compared to our present research, their study utilized a 1024 matrix with a constant pixel dimension of 0.34 × 0.34 mm^2^, which was slightly larger than our pixel size of 0.32 × 0.32 mm^2^. Milos et al. applied a QIR level of 3 based on data published by Sartoretti et al. [18]. However, both of those earlier studies were conducted using a previous software version of the scanner (VA40A) and not VA50A, with Sartoretti et al. using version VA40A and Milos et al. using the version VA40A service pack 1. The introduction of this service pack was accompanied by a reconfiguration in the naming sequence, where the previous QIR-2 level was redesignated as QIR-1; the former QIR-3 was retitled as QIR-2, and so on. Additionally, the new QIR-4 level was introduced. According to this new nomenclature, QIR-2 was favored in the study by Sartoretti et al., and QIR-3 was applied by Milos et al. Their use of a lower QIR level might explain why a much higher radiation dose was applied in the study by Milos et al. [19]; they applied an IQ level of 80 (we used a level of 13). This resulted in a median CTDI_Vol_ of 5.2 mGy, which was seven times higher than the median CTDI_Vol_ in our present study.

In the study by Sartoretti et al. [18], which favored the QIR-2 level (according to the current naming convention), they also applied a low-dose protocol with an IQ level of 15. However, their study utilized a standard protocol with 140 × 0.4 mm collimation, 1.5 mm slice thickness, and a 512 × 512 matrix. The resulting pixel sizes were not explicitly mentioned but are estimated to be around 0.7 × 0.7 mm^2^. As we hypothesized, this standard HR recommendation for the QIR level should not be directly adopted for UHR protocols. Our present results suggested that the highest QIR level (QIR-4) was most favorable for UHR-PCD-CT of the lungs. This is consistent with previous PCD-CT studies, which also recommend the highest QIR level for coronary CT angiography [24,25].

Several studies have demonstrated the superiority of PCD-CT over EID-CT for low-dose HRCT [9,16,26,27]. They have reported preserved or better image quality, with significantly reduced radiation doses, for PCD-CT operated in the standard mode (beam collimation, 140 × 0.4 mm). The radiation doses used in our low-dose UHR-PCD-CT study were within the same range. As recently published, the UHR mode (120 × 0.2 mm) of the PCD-CT scanner allows for dose reduction compared to the standard mode (140 × 0.4 mm) if images are reconstructed with the same slice thickness, which is based on the small pixel effect [28]. Because the detector elements are smaller in the fan direction, the small pixel effect enables superior image quality in ultra-low-dose examinations. Nevertheless, in this low-dose range, images with a 0.2 mm slice thickness are disturbed by increasing noise levels. This might be improved by the application of higher radiation doses.

In our low-dose study, although we observed improved image quality in the submillimeter (0.4 mm) reconstructions, we did not find the expected improvement in diagnostic ability. This might have been because the clinical reference images with 1.0 mm slice thickness were still in ultra-high-resolution since all images were reconstructed using a 1024 matrix. Another reason might be the low-dose protocol. Previous studies reporting improvements in UHR-PCD-CT for the diagnosis of interstitial lung diseases (ILD) have applied higher radiation doses. For example, a study of rheumatoid-arthritis ILD used a median CTDI_Vol_ of 8.18 mGy in the UHR-PCD-CT scans, with 0.2 mm slice thickness reconstructions [29]. However, for screening purposes, a low-dose UHR-PCD-CT protocol should be applied, as described in our study, which can be complemented by a higher-dose protocol in selected cases. For example, subtle interstitial changes in early ILD/interstitial lung abnormalities might require a minimum radiation dose to provide high diagnostic accuracy [29,30,31].

Lung cancer screening is being introduced in a rising number of countries. A low-dose UHR protocol is favored, especially in the context of this screening application, which requires a great increase in the number of lung examinations for the population. This protocol enables minimal dose exposure for potentially healthy individuals in the screening program while still providing satisfactory image quality to ensure high diagnostic reliability.

In addition to the improved detectability of well-known pulmonary pathologies, UHR-CT has the potential to reveal discreet anatomical alterations that enable earlier and more precise detection of lung diseases. This feature might be accessible through improved radiomics features with better cluster separation [32,33,34].

The present study has several limitations. Notably, it was a single-center investigation. Additionally, some lung pathologies had only a small sample size, and only two submillimeter slice thicknesses were evaluated. Also, the heterogeneity of the patient collective limits the results to a general reconstruction protocol. Specific clinical pictures could possibly benefit from deviating reconstruction parameters examined with higher radiation doses. Another limitation is that the diagnostic ability was only estimated and not measured based on the readers’ sensitivity and specificity. Finally, the images were evaluated relative to the clinical reference, and not all possible pairwise comparisons were analyzed.

In conclusion, our present results obtained from a small number of patients provide an optimized reconstruction protocol for low-dose ultra-high-resolution PCD-CT of the lungs for the first time in the field. These images scanned with a CTDI_vol_ around 0.7 mGy should be reconstructed with a 1024 matrix, a slice thickness of 0.4 mm, the sharpest lung kernel (Bl64), and the highest QIR level (QIR-4). Applying these optimized reconstruction settings can enable the full potential of low-dose UHR-PCD-CT for future applications.

## Figures and Tables

**Table 1:** Technical data for the computed tomography (CT) protocol and image reconstruction parameters.

Software version	syngo CT VA50A
Beam collimation	120 × 0.2 mm
Spiral pitch factor	0.8
Rotation time	0.25 s
Tube voltage	100 kVp
Tin filter	on
Tube current modulation	CARE Dose4D
Image quality level	13
Convolution kernel	Bl64
Slice thickness	0.2 mm, 0.4 mm, and 1.0 mm
Increment	0.15 mm, 0.3 mm, and 0.8 mm
Iterative reconstruction	QIR-2, QIR-3, and QIR-4

QIR: quantum iterative reconstruction.

**Table 2:** Baseline characteristics and radiation doses.

Numbers of patients	*n* = 51
Age, years	64 (54.5–72.5)
Sex	
Female	20 (39%)
Male	31 (61%)
Body height, cm	174 ± 10
Body weight, kg	76 ± 14
Body mass index, kg/m^2^	25.1 ± 4.2
CTDI_Vol_, mGy	0.74 ± 0.16
DLP, mGy × cm	23.9 (21.7–26.9)
Effective dose, mSv	0.34 (0.31–0.39)

Values are presented as mean ± standard deviation or median (interquartile range).

**Table 3:** Clinical indications for computed tomography (CT) lung scans.

Clinical Indications	Patients, *n* (%)
Pneumonia or therapy-related pneumonitis, diagnostic evaluation	10 (20%)
Pneumonia or therapy-related pneumonitis, follow-up	8 (16%)
Pneumonia exclusion before allogeneic stem cell transplantation	3 (6%)
Exclusion of pulmonary graft-versus-host disease after allogeneic stem cell transplantation	8 (16%)
Pulmonary nodules, diagnostic evaluation	7 (14%)
Pulmonary nodules, follow-up	8 (16%)
Pulmonary involvement of a rheumatic disease	5 (10%)
Others	2 (4%)

**Table 4:** Median noise levels of different reconstructions.

Slice Thickness (mm)	QIR Level	Noise Level (HU)
0.2	2	240 (220–270)
	3	180 (160–200)
	4	120 (100–130)
0.4	2	160 (150–180)
	3	120 (110–140)
	4	80 (72–92)
1.0	2	110 (100–130)
	3	84 (76–93)
	4	55 (50–62)

Interquartile ranges are given in parentheses.

**Table 5:** Pathologies evaluated in the qualitative analysis.

Pathology	Patients, *n* (%)
Number of lung nodules	
1	11 (22%)
2	7 (14%)
≥3	16 (31%)
Bronchial pathologies	7 (14%)
Emphysema and bullae	14 (27%)
Ground-glass opacities	30 (59%)
Mosaic pattern	6 (12%)
Interstitial abnormalities	19 (37%)
Pleural effusion	12 (24%)

## Data Availability

Data cannot be shared publicly because of institutional and national data policy restrictions since the data contain potentially identifying patient information. The datasets used and/or analyzed during the current study are available from the corresponding author upon a reasonable request.
